# Supplementary material for: Using youth-engaged research methods to develop a measure of disordered eating in transgender, non-binary, and gender-diverse youth: Research protocol
Source: PLoS One. 2024 Nov 20;19(11):e0313908. doi: 10.1371/journal.pone.0313908 (PMC11578474; doi:10.1371/journal.pone.0313908)
Supplement: Attachment — Submitted filename: PLOS ONE Peer Review.pdf [file pone.0313908.s001.pdf]

Abstract:
Transgender, non-binary, and gender-diverse (TNG) youth experience disordered eating behaviors (DEBs) for reasons unique to their gender identity

- **I recommend shifting this to have the onus be placed on the treatment of TNG youth, internalized gendered appearance ideals, internalized cisheteronormativity, etc. A TNG individual's unique gender identity is in itself not inherently a risk factor.**

Although the field of TNG DEBs research is quickly growing, gaps in knowledge remain, due, in part, to a lack of TNG input on research protocols focused on this population, and underrepresentation of TNG participation in research.

- **This is an excellent point. I would encourage expanding on this briefly to indicate how the lack of TNG input frequently translates into flawed methodologies. It feels loosely inferred in lines 62-71, but stating this more explicitly may be more impactful.**

Introduction:
TNG youth with eating disorder symptomatology are at the intersection of multiple, life-threatening risk factors.

- **This sentence is a bit circular - risk factors for what? Throughout the paper, there is little to no mention of DEBs resulting in physical health impairments. The closing sentence of this paragraph mentions multiple health disparities. Perhaps this is a good place to introduce content about that.**

No current measures assess the attitudes and cognitions underlying an individual TNG youth's DEBs. This creates a major barrier to culturally specific care. A tailored instrument that assesses the specific behaviors, attitudes, and cognitions contributing to TNG youths' DEBs is needed to advance research on this understudied topic and improve clinical care.

- **I would encourage mention of underdiagnosis specifically, as it is most directly and immediately related to creating a screening and/or assessment tool.**

The paragraph discussing underrepresentation of TNG participants in research and barriers to participation (lines 43-61) may be strengthened by including a mention of state surveillance and safety risks of disclosure. This is very briefly mentioned in lines 253-255, but merits being introduced earlier. Also consider highlighting rural and/or southern TNG youth as potentially being underrepresented in research – confirm with a literature search.

In addition to confirming scale structure, measurement invariance in relation to sex assigned at birth, gender identity, and race/ethnicity will be evaluated. Based on extant literature, we will also explore associations between DEBs and TNG-specific risk and protective factors (i.e., mental health diagnoses, gender minority stress, peer/family support, access to affirming medical care)

- **Have you considered incorporating socioeconomic status? There is some great research highlighting the link between food insecurity and DEBs, and showing increased food insecurity in TNG populations.**

Compensation for YAB is mentioned in line 268. However, it is mentioned that compensation will occur quarterly, while elsewhere in the paper, it reads that YAB will meet twice monthly. Please provide a rationale for this and detail your plan for compensating research participants outside of the YAB.

Sociodemographic survey: questions assessing an individual's age, sex assigned at birth, gender identity, race/ethnicity, medical affirmation (gender-affirming medications and surgeries), congruence of gender identity and expression, history of mental health and eating disorder diagnoses

- **Please consider including intersex experience in this study and account for how you may do that.**

Using a data-driven, inductive approach, the research team will work with the YAB members who are 16-17 years old to establish themes (Step 3). The PI and research assistant will meet with all YAB members biweekly to review patterns within the data and identify persistent themes (Steps 4-5).

- **Please clarify how this will work. Will a portion of the group establish themes, and then the full group will establish themes again? Is this meant to provide interrater reliability?**

This multi-phase study protocol applies youth-engaged research methods and uses inclusive, affirming research processes and will result in: 1) the first study to explore both barriers and motivators to health research in TNG youth,

- **I caution against claiming to be the first to any authors. I encourage reading through some of these papers and reassessing language:**
    - Self-Consent for HIV Prevention Research Involving Sexual and Gender Minority Youth: Reducing Barriers Through Evidence-Based Ethics
    - Lessons from a community-based participatory research study with transgender and gender nonconforming youth and their families
    - Researching and Working for Transgender Youth: Contexts, Problems and Solutions
    - A scoping review of good methodological practices in research involving transgender, non-binary, and two-spirit youth
    - The Inclusion of the LGBTQIA+ Community in Research: a Rapid Scoping Review on Barriers and Facilitators
    - Perceptions of Barriers to and Facilitators of Participation in Health Research Among Transgender People

In line 260, “our samples will not be skewed heavily towards white, transmasculine youth receiving gender-affirming care”

- **Consider rephrasing to show what you *are* doing and avoid putting too much emphasis on white transmasculine people in the paper. It’s a good point, but also a point that when articulated in particular ways, seem to position white, transmasculine youth as problematic instead of research methodology, recruitment, distrust, etc. ,**

Line 276, ”reducing mental health disparities of TNG youth”

- **Consider including physical health disparities that result from DEBs.**

There is no conclusion section; I recommend turning the last paragraph into a conclusion.
