# Supplementary material for: Using youth-engaged research methods to develop a measure of disordered eating in transgender, non-binary, and gender-diverse youth: Research protocol
Source: PLoS One. 2024 Nov 20;19(11):e0313908. doi: 10.1371/journal.pone.0313908 (PMC11578474; doi:10.1371/journal.pone.0313908)
Supplement: Attachment — Submitted filename: PlosOne_TNGED.docx [file pone.0313908.s002.docx]

**Overall Comments:**

This is an exciting study that addresses an important gap in the literature and the authors intend to maximize TNG youth involvement. However, important details are missing processes for community engagement and how compensation is determined, especially due to the amount of labor they are requesting from TNG youth. The methods for qualitative analysis are also underdeveloped and inconsistent with how Braun & Clarke’s approach to thematic analysis has evolved. There is no mention of reflexivity as part of the analytic process, which is particularly concerning considering the nature of the study. Citations throughout are also relatively old and many could be updated. Finally, more attention could be paid to language throughout. I am curious if excluding intersex youth from TNG is on purpose or an oversight. If the study intends to include intersex youth please modify sociodemographic information collected.

**Abstract**

Line 5- “for reasons unique to their gender identity”- This phrase is misleading, this insinuates that eating disorders are inherent to gender identity rather due to larger factors such as discrimination, marginalization, Euro-centric gendered beauty ideals, and lack of access to gender-affirming medical care. I recommend revising.

Line 7- Recommend removing “vulnerable”—vulnerable to what? TNG youth are also inspiring, determined, and strong. This language is not helpful and does not add to this sentence.

Line 10- TNG populations are generally excited to participate in research with research institutions/individuals that they trust because they recognize TNG people have been excluded from research and much needs to be done to improve care for TNG people. Hesitations to engage in research are typically due to fear for safety/confidentiality which is not discussed here. It also may be helpful to specify ED research rather than speaking to research overall. See Burnette et al., 2022 re: demographics included in eating disorder treatment trials.

**Introduction**

Line 20-22- It is important to mention that vulnerability to body dissatisfaction among youth may also be exacerbated due to lack of access to gender-affirming medical interventions. Currently this is only mentioned in the discussion. The way this is written now it makes it seem like DEBs among TNG youth is an inherent part of pubertal development. Also, there is no mention of economic disparities (due to structural discrimination/marginalization) among trans populations and how that impacts DEBs.

Line 27-28- you say multiple “life-threatening risk factors”, but only list suicidality?

Line 31- remove “significantly increased”—this is redundant based on prior sentence.

Line 52- accurate is said twice in this sentence, I recommend removing the first “accurate”

Line 55-56- this is old data (particularly Arcelus), I’d recommend updating citation or mentioning here that recent estimates are not available. It seems odd to me to focus so much on percentages of trans masc vs trans femme vs nonbinary/gender diverse, especially as these categories are not exclusive, for example many people are trans masc or trans femme and nonbinary. It is important to focus on transfemme recruitment due to underrepresentation but I would not make it as much of a central point, especially because the data cited is not recent and there have been evolutions in how we collect gender identity information.

Line 59- these citations are also old (2006-2009), there have been many studies about health disparities among trans populations published in the past five years. One example is: Hughes et al., 2022- which looks at mortality rates.

Line 69- “culturally specific” could be deleted here. Overall “culturally specific” is used frequently. However, it is not defined what this means. There are many cultures within TNG communities (stated in the following sentence), so this use throughout without defining what is meant by the term “cultural” is strange and communicates assumptions about monolithic trans experience.

Line 70-71- Why is trans femme excluded here? When the authors spent a while talking about trans femme exclusion earlier in the introduction?

**Methods**

Line 78- isn’t it disordered eating behaviors *and* related attitudes and cognitions? Something just assessing DEB prevalence would be a different measure and this study’s interest in attitudes and cognitions underlying DEBs is unique. I would recommend being consistent throughout.

Line 79-80- how will the YAB be recruited? I see that later they will be compensated quarterly, but how is compensation determined? This is a lot of labor for a YAB.

Line 86- Thematic saturation may not be a feasible goal (or a helpful metric) for this type of thematic analysis. See: Braun V, Clarke V. To saturate or not to saturate? Questioning data saturation as a useful concept for thematic analysis and sample-size rationales. *Qualitative Research in Sport, Exercise and Health*. 2021;13(2):201-216. doi:[10.1080/2159676X.2019.1704846](https://doi.org/10.1080/2159676X.2019.1704846)

Line 90-94- Have the authors considered any particular facilitation strategy for these meetings? Co-design strategies or concept mapping are some examples of facilitation strategies designed to maximize meaningful engagement with YABs. Have facilitators received any training on YAB facilitation?

Line 97- throughout “patterns of cognitive processing problems” is used. This reads as pathologizing rather than indicating issues with the measure itself. You could just say patterns of cognitive processing rather than naming it as problems.

Line 115- Recruitment- It felt odd to put recruitment information after the stages, I would think it would make more sense to put recruitment information upfront or integrated throughout the Phases

Line 141- How will history of DEBs be assessed for those participating in the advisory board?

**Data collection**

Overall comments. This is a lot of work from a YAB. I like the plan to continuously get feedback from members about how to maximize involvement reduce barriers, but the lack of info re: compensation is concerning.

**Analyses**

If it was an intentional decision to not use recent literature about thematic analyses, please state that reason here. It seems particularly important when doing qualitative research with gender diverse and racial/ethnically diverse populations to be using a *reflexive* thematic approach, which is not mentioned at all throughout this entire protocol. I recommend reviewing

Braun V, Clarke V. *Thematic Analysis: A Practical Guide*. SAGE; 2022.

This study says it uses qualitative methods but relies on quantitative metrics to demonstrate rigor, without any critique of the limitations of quantitative metrics to evaluate qualitative research. See chapter 8 of the book listed above (Braun & Clarke, 2022). Why are the authors using thematic analysis when they are committed to using quantitative measures to assess rigor? Another qualitative method that is more in alignment with the authors framework may be a better fit for this study.

**Discussion**

Citations are lacking throughout the discussion and there is limited engagement with literature about developing YABs and procedures for conducing youth-engaged research. This study may overstate its novelty and does not reference any other examples of how YABs have been used in related fields.

How will this measure be made available for other clinicians and researchers?
