# Supplementary material for: Using youth-engaged research methods to develop a measure of disordered eating in transgender, non-binary, and gender-diverse youth: Research protocol
Source: PLoS One. 2024 Nov 20;19(11):e0313908. doi: 10.1371/journal.pone.0313908 (PMC11578474; doi:10.1371/journal.pone.0313908)
Supplement: Attachment — Submitted filename: Response to Reviewers.docx [file pone.0313908.s003.docx]

Thank you for taking the time to provide thoughtful reviews of our manuscript, “*Using youth-engaged research methods to develop a measure of disordered eating in transgender, non-binary, and gender-diverse youth: research protocol*”. We have made edits based on the feedback provided and it has strengthened this manuscript. Here is our response to each item of feedback organized by manuscript section. All referenced lines correspond to the line numbers in the **unmarked version** of our revised paper.

**ABSTRACT**

1. Reviewer #1: “Transgender, non-binary, and gender-diverse (TNG) youth experience disordered eating behaviors (DEBs) for reasons unique to their gender identity” I recommend shifting this to have the onus be placed on the treatment of TNG youth, internalized gendered appearance ideals, internalized cisheteronormativity, etc. A TNG individual’s unique gender identity is in itself not inherently a risk factor. Additionally from Reviewer #2: Line 5- “for reasons unique to their gender identity”- This phrase is misleading, this insinuates that eating disorders are inherent to gender identity rather due to larger factors such as discrimination, marginalization, Euro-centric gendered beauty ideals, and lack of access to gender-affirming medical care. I recommend revising.
   a. We thank both reviewers for their thoughtful recommendations and we agree that risk factors for DEBs in TNG youth are not inherent to gender identity, or even gender dysphoria, but external factors placed on TNG youth. The sentence (lines 4-7) now reads, “Transgender, non-binary, and gender-diverse (TNG) youth experience disordered eating behaviors (DEBs) for reasons unique to their sociocultural positioning and the specific challenges they face, including societal beauty expectations of gender, transphobic discrimination, and lack of access to gender affirming medical care.”
2. Reviewer #2: Line 7- Recommend removing “vulnerable”—vulnerable to what? TNG youth are also inspiring, determined, and strong. This language is not helpful and does not add to this sentence.
   a. We appreciate the reviewer’s thoughtfulness around affirming and supportive language when discussing TNG youth. We have deleted this word from the sentence in line 9.
3. Reviewer #2: Line 10- TNG populations are generally excited to participate in research with research institutions/individuals that they trust because they recognize TNG people have been excluded from research and much needs to be done to improve care for TNG people. Hesitations to engage in research are typically due to fear for safety/confidentiality which is not discussed here. It also may be helpful to specify ED research rather than speaking to research overall. See Burnette et al., 2022 re: demographics included in eating disorder treatment trials.
   a. We have added “DEBs research” to the end of this sentence (lines 9-12) to specify that these are gaps in this research area.
   b. Lines 10-13 provide a summary of gaps in research. We provide greater details on underrepresentation of TNG participants in DEBs research in the 3^rd^ paragraph of the introduction section.

**INTRODUCTION**

1. Reviewer #1: “TNG youth with eating disorder symptomatology are at the intersection of multiple, life-threatening risk factors.” This sentence is a bit circular - risk factors for what? Throughout the paper, there is little to no mention of DEBs resulting in physical health impairments. The closing sentence of this paragraph mentions multiple health disparities. Perhaps this is a good place to introduce content about that. Additionally, Reviewer #2: Line 27-28- you say multiple “life-threatening risk factors”, but only list suicidality?
   a. We have deleted this sentence completely given multiple reviewers noted that it was confusing. We also added examples of physical and emotional impairments. This section (lines 28-35) now reads, “Based upon data from the general population, DEBs are associated with significant impairment (i.e., changes in menstruation and fertility, substance use, depressive symptoms) and often precede the onset of eating disorders. Furthermore, 30-51% of TNG youth report suicidal ideation, and TNG young adults with eating disorders attempt suicide at higher rates than their TNG peers without eating disorders. Despite these health disparities among TNG youth, DEBs and eating disorder research has historically focused on white, cisgender young adult women.”
2. Reviewer #1: “No current measures assess the attitudes and cognitions underlying an individual TNG youth’s DEBs. This creates a major barrier to culturally specific care. A tailored instrument that assesses the specific behaviors, attitudes, and cognitions contributing to TNG youths’ DEBs is needed to advance research on this understudied topic and improve clinical care.” I would encourage mention of underdiagnosis specifically, as it is most directly and immediately related to creating a screening and/or assessment tool.
   a. We agree with the reviewer and these sentences (lines 41-43) now read, “No current measures assess the attitudes and cognitions underlying an individual TNG youth’s DEBs. This creates a major barrier to gender-affirming care and accurate diagnosis of EDs and DEBs in trans youth.”
3. Reviewer #1: “Although the field of TNG DEBs research is quickly growing, gaps in knowledge remain, due, in part, to a lack of TNG input on research protocols focused on this population, and underrepresentation of TNG participation in research.” This is an excellent point. I would encourage expanding on this briefly to indicate how the lack of TNG input frequently translates into flawed methodologies. It feels loosely inferred in lines 62-71, but stating this more explicitly may be more impactful.
   a. Thank you for the opportunity to provide more details on this topic. We have added the sentences (lines 64-67), “Race/ethnicity, access to resources, and gender identity have an impact on TNG youth experiences. As a result, if researchers are unable to recruit samples that represent TNG youth diversity, validity can be compromised”
4. Reviewer #1: The paragraph discussing underrepresentation of TNG participants in research and barriers to participation (lines 43-61) may be strengthened by including a mention of state surveillance and safety risks of disclosure. This is very briefly mentioned in lines 253-255, but merits being introduced earlier. Also consider highlighting rural and/or southern TNG youth as potentially being underrepresented in research – confirm with a literature search.
   a. We have added lines 52-53, “Moreover, the increase in anti-transgender legislation over the past 5 years may affect TNG youth research participation.”
   b. In line 56, we now specify oversampling of TNG youth with access to gender-affirming care in urban areas.
5. Reviewer #2: Line 20-22- It is important to mention that vulnerability to body dissatisfaction among youth may also be exacerbated due to lack of access to gender-affirming medical interventions. Currently this is only mentioned in the discussion. The way this is written now it makes it seem like DEBs among TNG youth is an inherent part of pubertal development. Also, there is no mention of economic disparities (due to structural discrimination/marginalization) among trans populations and how that impacts DEBs.
   a. We agree that access to gender-affirming care may affect body dissatisfaction associated with pubertal changes. This sentence (lines 22-26) has been modified to, “TNG youth also have increased vulnerability to body dissatisfaction due to pubertal changes and development of secondary sexual characteristics that might be misaligned with their gender identity, which may be exacerbated by a youth’s inability to access gender-affirming medical care (i.e., puberty blockers, gender-affirming hormones).”
   b. We only found one article describing socioeconomic status and DEBs/eating disorders and it is a qualitative study (https://pubmed.ncbi.nlm.nih.gov/39102353/). We have added measures of socioeconomic status to Phase 4 surveys to better understand this relationship.
6. Reviewer #2: Line 31- remove “significantly increased”—this is redundant based on prior sentence.
   a. We have deleted “significantly increased” from this sentence (lines 33-35).
7. Reviewer #2: Line 52- accurate is said twice in this sentence, I recommend removing the first “accurate”
   a. Thank you for the opportunity to correct this error. We have deleted the first “accurate” in this sentence (lines 58-64).
8. Reviewer #2: Line 55-56- this is old data (particularly Arcelus), I’d recommend updating citation or mentioning here that recent estimates are not available. It seems odd to me to focus so much on percentages of trans masc vs trans femme vs nonbinary/gender diverse, especially as these categories are not exclusive, for example many people are trans masc or trans femme and nonbinary. It is important to focus on transfemme recruitment due to underrepresentation but I would not make it as much of a central point, especially because the data cited is not recent and there have been evolutions in how we collect gender identity information.
   a. Thank you for the opportunity to provide more up to date estimates of TNG people by gender identity. We have provided estimates from a 2022 report (Reference #25).
   b. We agree that measuring gender identity is a complex process and we describe issues with estimates in the sentence (lines 57-64), “Approximately 38.5% of TNG adults are transfeminine, 35.9% transmasculine, and 25.6% gender non-conforming[24]. Although estimates of TNG population are not fully accurate due to the broad range of terms surveys use to describe gender identity, lack of TNG data collection in population-based surveys, and difficulty creating gender identity categories (as individuals can identify with multiple gender identities), this discrepancy between the proportion of transfeminine people and their underrepresentation in research is a critical gap in knowledge because transfeminine people, particularly transfeminine people of color, experience unique physical and mental health challenges.” We acknowledge that research has evolved with respect to measurement of gender identity in research, and we are only able to report proportions based on the individual study’s methods even if outdated at present. We have chosen to keep this information in the manuscript because we feel it is important to describe the difference in representation of gender identities in research samples versus population estimates of gender identity.
9. Reviewer #2: Line 59- these citations are also old (2006-2009), there have been many studies about health disparities among trans populations published in the past five years. One example is: Hughes et al., 2022- which looks at mortality rates.
   a. Thank you for directing our attention to more recent literature. We now include more recent citations, such as the Hughes 2022 article recommended by the reviewer (References #26-29).
10. Reviewer #2: Line 69- “culturally specific” could be deleted here. Overall “culturally specific” is used frequently. However, it is not defined what this means. There are many cultures within TNG communities (stated in the following sentence), so this use throughout without defining what is meant by the term “cultural” is strange and communicates assumptions about monolithic trans experience.
    a. Thank you for this thoughtful recommendation. We have deleted “culturally specific” and “culturally sensitive” throughout the manuscript.
11. Reviewer #2: Line 70-71- Why is trans femme excluded here? When the authors spent a while talking about trans femme exclusion earlier in the introduction?
    a. We have added transfeminine individuals to this sentence (lines 76-79).

**MATERIALS AND METHODS**

Methods

1. Reviewer #1: Compensation for YAB is mentioned in line 268. However, it is mentioned that compensation will occur quarterly, while elsewhere in the paper, it reads that YAB will meet twice monthly. Please provide a rationale for this and detail your plan for compensating research participants outside of the YAB.
   a. Thank you for the opportunity to correct this error and add these details. We have added a column in Table #2 that provides the compensation amount for each research activity.
2. Reviewer #1: “In addition to confirming scale structure, measurement invariance in relation to sex assigned at birth, gender identity, and race/ethnicity will be evaluated. Based on extant literature, we will also explore associations between DEBs and TNG-specific risk and protective factors (i.e., mental health diagnoses, gender minority stress, peer/family support, access to affirming medical care).” Have you considered incorporating socioeconomic status? There is some great research highlighting the link between food insecurity and DEBs, and showing increased food insecurity in TNG populations.
   a. We thank the reviewer for this important recommendation. We have added measures of socioeconomic status (education, income, food insecurity) to the data that will be collected in Phase 4.
3. Reviewer #2: Line 78- isn’t it disordered eating behaviors *and* related attitudes and cognitions? Something just assessing DEB prevalence would be a different measure and this study’s interest in attitudes and cognitions underlying DEBs is unique. I would recommend being consistent throughout.
   a. We have edited the sentence (lines 84-85) to read, “This research protocol will use a community-engaged approach to create a TNG youth-centered measure of disordered eating behaviors, attitudes, and cognitions.”
4. Reviewer #2: Line 79-80- how will the YAB be recruited? I see that later they will be compensated quarterly, but how is compensation determined? This is a lot of labor for a YAB.
   a. Recruitment details for the YAB can be found in lines 159-165.
   b. Thank you for the opportunity to provide clarifying information on YAB compensation. We have added lines 87-94 to describe the responsibilities and time commitment for active participation as a YAB member.
5. Reviewer #2: Line 86- Thematic saturation may not be a feasible goal (or a helpful metric) for this type of thematic analysis. See: Braun V, Clarke V. To saturate or not to saturate? Questioning data saturation as a useful concept for thematic analysis and sample-size rationales. *Qualitative Research in Sport, Exercise and Health*. 2021;13(2):201-216. doi:10.1080/2159676X.2019.1704846
   a. We thank the reviewer for providing their expertise on thematic analysis. We have read the recommended reading and put more thought into our sample size for Phase 2. This section (lines 108-112) now reads, “We will oversample underrepresented TNG identities (i.e., TNG youth of color, transfeminine and non-binary youth). We have chosen a sample size of 20 participants based on the focus of the research question, methods of data collection (see below), sample diversity, and depth of data likely generated from each participant.”
6. Reviewer #2: Line 90-94- Have the authors considered any particular facilitation strategy for these meetings? Co-design strategies or concept mapping are some examples of facilitation strategies designed to maximize meaningful engagement with YABs. Have facilitators received any training on YAB facilitation?
   a. We thank the reviewer for the opportunity to expand on our facilitation approach to YAB participation/meetings. We have added lines 94-104.
7. Reviewer #2: Line 97- throughout “patterns of cognitive processing problems” is used. This reads as pathologizing rather than indicating issues with the measure itself. You could just say patterns of cognitive processing rather than naming it as problems.
   a. Thank you to the reviewer for their dedication to affirming language. We have deleted “problems” from line 126.
8. Reviewer #2: Line 115- Recruitment- It felt odd to put recruitment information after the stages, I would think it would make more sense to put recruitment information upfront or integrated throughout the Phases
   a. In the current format, we provide a summary of each phase at the beginning of the Materials and methods section and then have the subsections: recruitment, inclusion/exclusion, data collection, and analyses; starting with recruitment. Because there are multiple phases and similar processes across different phases in all subsections (i.e., similar recruitment practices for Phases 2-4, similar qualitative data collection and analyses in Phases 2 and 3), we organized the Materials and methods section by these subsections instead of Phases to eliminate redundancy.
9. Reviewer #2: Line 141- How will history of DEBs be assessed for those participating in the advisory board?
   a. Thank you for the opportunity to include this information. We have added lines 177-185 under Table 1 which detail the inclusion criteria of history of DEBs for Phases 1 and 3a.
10. Reviewer #3: Although Phase 2 is an important aspect of the study, it seems to be less connected to the other phases. Is there a reason the authors are interested in general barriers to TNG youth participation in research and not barriers to research for TNG youth with disordered eating? Might TNG youth experiencing DEBs have additional or specific barriers to participation that TNG youth without disordered eating may not have that would not be captured in Phase 2?
    a. Phase 2 is specific and unique because the aim has broader application than just TNG youth with DEBs. We also agree with the reviewer that this phase should also give participants the opportunity to remark on any barriers and facilitators to research specific to DEBs research. Phase 2 inclusion criteria will not include a history of DEBs and the interview guide will include general questions on research participation, questions on participation in specific types of research (i.e., community-engaged, survey, and qualitative research), and questions specific to participation in research on DEBs. We have added lines 112-116 and lines 186-189 to provide these clarifying details.
11. Reviewer #3: The authors discuss some barriers to research participation for TNG youth, particularly parental consent and non-supportive caregivers. Given the high risk of safety concerns for TNG youth that may be outed by any potential breach of confidentiality, the authors are asked to address how they plan to safeguard participant identity and anonymity, particularly during qualitative interviews.
    a. Thank you for this important recommendation. We have added (lines 211-214) “The interviewer will confirm that the TNG youth participant is in a private and comfortable location before initiating any study-related conversations. We will obtain permission and preference of contact by phone or email when scheduling the interview” to the 2^nd^ paragraph of the data collection subsection.
12. Reviewer #3: The authors are encouraged to discuss any established plans for future changes to the study protocol after the YAB is established and how input from the YAB on study design will be solicited and incorporated prior to moving forward with Phases 2-4.
    a. We have added (lines 103-104) “This study protocol provides a foundation for this multi-phase study and will be modified accordingly as YAB members provide feedback throughout Phases 2-4”. Lines 94-101 and 198-209 provide details on our strategy for collecting input from YAB members.

Inclusion and exclusion criteria

1. Reviewer #3: I would encourage the authors to consider the age range for inclusion criteria for the study in Phases 2-4 and/or further explain the rationale for the chosen upper age limit. Because much of the introduction is specifically focused on TNG minors and many DEB scales begin at 18 for adult versions, it is unclear how the limit of 21 was chosen in how the authors define “youth”.
   a. Our inclusion criteria include TNG individuals 18-21 because studies show a peak in eating disorders during adolescence and young adulthood. We have added lines 192-193 to provide this explanation.

Data collection

1. Reviewer #1: “Sociodemographic survey: questions assessing an individual’s age, sex assigned at birth, gender identity, race/ethnicity, medical affirmation (gender-affirming medications and surgeries), congruence of gender identity and expression, history of mental health and eating disorder diagnoses.” Please consider including intersex experience in this study and account for how you may do that.
   a. We have added intersex to the sociodemographic survey. We will measure this by using InterACT’s recommendations (https://interactadvocates.org/) for intersex data collection. We have cited their website in the reference section (reference #53).
2. Reviewer #2: Overall comments. This is a lot of work from a YAB. I like the plan to continuously get feedback from members about how to maximize involvement reduce barriers, but the lack of info re: compensation is concerning.
   a. We agree that this information is important. We have added the compensation amount to Table 2. We have also added lines 87-94 to provide more details on YAB responsibilities and time commitment.
3. Reviewer #3: Given community-based recommendations against collecting sex assigned at birth from trans participants unless absolutely necessary, the authors are asked to consider how they will collect demographic data and the extent to which sex assigned at birth versus gender is necessary for ensuring measurement invariance.
   a. Thank you for this thoughtful comment. We agree that sex assigned at birth should only be collected when it is necessary for the research question. We are only collecting sex assigned at birth for Phase 4. We have included this in Phase 4 because we want to determine whether the measure is consistent (or not) across people born with different body parts (i.e., capacity to menstruate). We have added this specific example to line 276.
4. Reviewer #3: Since the authors are planning to ask about medical affirmation during the sociodemographic survey, they are also encouraged to ask about participants’ desire to access such medical care. Not all trans and genderqueer individuals desire or seek out gender affirming medical care, so it may be important to understand medical affirmation in the context of each participant’s desire for such care.
   a. We thank the reviewer for this important point. We have added desire for medical affirmation to the sociodemographic section of Phase 4 data collection.

Analyses

1. Reviewer #1: “Using a data-driven, inductive approach, the research team will work with the YAB members who are 16-17 years old to establish themes (Step 3). The PI and research assistant will meet with all YAB members biweekly to review patterns within the data and identify persistent themes (Steps 4-5).” Please clarify how this will work. Will a portion of the group establish themes, and then the full group will establish themes again? Is this meant to provide interrater reliability? Additionally, Reviewer #2: This study says it uses qualitative methods but relies on quantitative metrics to demonstrate rigor, without any critique of the limitations of quantitative metrics to evaluate qualitative research. See chapter 8 of the book listed above (Braun & Clarke, 2022). Why are the authors using thematic analysis when they are committed to using quantitative measures to assess rigor? Another qualitative method that is more in alignment with the authors framework may be a better fit for this study.
   a. Thank you for the opportunity to reflect on our qualitative analyses plan. After further discussions within our research team, we have decided that all YAB members, paired with research staff, will code and establish themes. Instead of conducting interclass correlations, each transcript will be coded by at least 2 coders, coders will meet bi-weekly to discuss any coding questions, and any discrepancies between coders will be resolved by the PI and research assistant who is trained in qualitative analyses and identifies as TNG. We have edited this section (lines 247-260), and it now reads, “The PI and research assistant will train YAB members to participate in reflexive thematic analysis. All TNG youth members, each paired with trained research staff, will code transcripts. Coders will independently read two transcripts to establish familiarity (Step 1). Coders will then use the first five transcripts to develop a codebook by independently coding the same transcripts, comparing results, and adjusting code definitions as needed (Step 2). Once a codebook is developed, each transcript will be coded by at least 2 YAB members (with the assistance of their research team partner). The research team will compare codes for each transcript and all discrepancies will be resolved by the PI and research assistant. Coders will meet with the PI and research assistant weekly to bi-weekly by Zoom video conference to discuss any questions on codes, code definitions, and coding process. Using a data driven, inductive approach, the research team will work with the YAB members to review patterns and establish themes (Step 3). The PI and research assistant will meet with YAB members bi-weekly to identify and confirm persistent themes (Steps 4-5).”
2. Reviewer #2: If it was an intentional decision to not use recent literature about thematic analyses, please state that reason here. It seems particularly important when doing qualitative research with gender diverse and racial/ethnically diverse populations to be using a *reflexive* thematic approach, which is not mentioned at all throughout this entire protocol. I recommend reviewing Braun V, Clarke V. *Thematic Analysis: A Practical Guide*. SAGE; 2022.
   a. Thank you for this important recommendation. We have reviewed this literature, updated our citations (reference #45), and applied this literature to modifications of the analysis section of our manuscript (lines 247-260).

**DISCUSSION**

1. Reviewer #1: “This multi-phase study protocol applies youth-engaged research methods and uses inclusive, affirming research processes and will result in: 1) the first study to explore both barriers and motivators to health research in TNG youth…” I caution against claiming to be the first to any authors. I encourage reading through some of these papers and reassessing language: Self-Consent for HIV Prevention Research Involving Sexual and Gender Minority Youth: Reducing Barriers Through Evidence-Based Ethics, Lessons from a community-based participatory research study with transgender and gender nonconforming youth and their families, Researching and Working for Transgender Youth: Contexts, Problems and Solutions, A scoping review of good methodological practices in research involving transgender, non-binary, and two-spirit youth, The Inclusion of the LGBTQIA+ Community in Research: a Rapid Scoping Review on Barriers and Facilitators, Perceptions of Barriers to and Facilitators of Participation in Health Research Among Transgender People
   a. Thank you for this thoughtful critique. We have edited this section (lines 282-287) to now read, “This multi-phase study protocol applies youth-engaged research methods and uses inclusive, affirming research processes and will explore both barriers and motivators to health research in TNG youth and apply these findings to recruit under researched subpopulations of TNG youth (i.e., transfeminine youth, TNG youth of color, and TNG youth without family support and/or access to gender-affirming medical care) in order to create a tailored, evidence-based DEBs measure for TNG youth.”
   b. Additionally, thank you for taking the time to compile this list of references. We have reviewed the articles, and one study in particular has provided great context for the second paragraph of our discussion section (reference #41).
2. Reviewer #1: In line 260, “our samples will not be skewed heavily towards white, transmasculine youth receiving gender-affirming care” consider rephrasing to show what you *are* doing and avoid putting too much emphasis on white transmasculine people in the paper. It’s a good point, but also a point that when articulated in particular ways, seem to position white, transmasculine youth as problematic instead of research methodology, recruitment, distrust, etc.
   a. We agree with the reviewer and this sentence (lines 308-313) now reads, “As a result, our study samples may not fully reflect the diversity of TNG youth; however, by: 1) including a research activity that explores the barriers and facilitators to research participation, 2) oversampling underrepresented subpopulations of TNG youth in all research phases, and 3) requesting a waiver of parental consent in Phases 2-4, we have made sample diversity a main priority throughout our entire multi-phase research protocol.”
3. Reviewer #1: Line 276, ”reducing mental health disparities of TNG youth”. Consider including physical health disparities that result from DEBs.
   a. We have added “physical health” to line 330.
4. Reviewer #1: There is no conclusion section; I recommend turning the last paragraph into a conclusion.
   a. The last paragraph is now a Conclusion section.
5. Reviewer #2: Citations are lacking throughout the discussion and there is limited engagement with literature about developing YABs and procedures for conducing youth-engaged research. This study may overstate its novelty and does not reference any other examples of how YABs have been used in related fields.
   a. Thank you for this recommendation. We have added additional citations throughout the Discussion section (references #41, 57-63).
6. Reviewer #2: How will this measure be made available for other clinicians and researchers?
   a. Thank you for the opportunity to provide dissemination information. The measure will be freely available. We have added lines 143-144.
