# Supplementary material for: Using youth-engaged research methods to develop a measure of disordered eating in transgender, non-binary, and gender-diverse youth: Research protocol
Source: PLoS One. 2024 Nov 20;19(11):e0313908. doi: 10.1371/journal.pone.0313908 (PMC11578474; doi:10.1371/journal.pone.0313908)
Supplement: Attachment — Submitted filename: Response to Reviewers.docx [file pone.0313908.s004.docx]

Thank you for taking the time to provide thoughtful reviews of our manuscript, “*Using youth-engaged research methods to develop a measure of disordered eating in transgender, non-binary, and gender-diverse youth: research protocol*”. We have made edits based on the feedback provided and it has strengthened this manuscript. Here is our response to each item of feedback organized by manuscript section. All referenced lines correspond to the line numbers in the **unmarked version** of our revised paper.

**OVERALL**

1. Reviewer #2: There are several areas where the language is not as concise as it could be (e.g. using "as a result of" instead of "due to", "not recreate" instead of "avoid"), and sentences are quite long/meandering e.g., lines 69-75 (could be changed to something like "although prevalence rates of gender diverse identities vary significantly across surveys, trans femme people are underrepresented in eating disorder research (cite). It is important to address this gap because..."). I'm also not sure about the language "biased towards" in line 173. I recognize that some of this is a style preference, but I do think that further editing could improve readability throughout.
   a. Thank you for your feedback. We have made the following edits:
      i. Replaced “as a result of” with “due to” in line 21.
      ii. Replaced “not recreate” with “avoid” in line 169.
      iii. Replaced “has been biased towards” with “has primarily sampled” in line 163.
      iv. Regarding the original sentence, “Although estimates of TNG population are not fully accurate due to the broad range of terms surveys use to describe gender identity, lack of TNG data collection in population-based surveys, and difficulty creating gender identity categories (as individuals can identify with multiple gender identities), this discrepancy between the proportion of transfeminine people and their underrepresentation in research is a critical gap in knowledge because transfeminine people, particularly transfeminine people of color, experience unique physical and mental health challenges[26-29].”, we did not simplify per the recommendation of the reviewer because we feel it is important that the reader learn about the reasons for inaccurate estimates. We agree with the reader that the original sentence was difficult to read because of its length and we have separated it into 2 separate sentences, “Estimates of TNG population are not fully accurate due to the broad range of terms surveys use to describe gender identity, lack of TNG data collection in population-based surveys, and difficulty creating gender identity categories (as individuals can identify with multiple gender identities). Despite this, the discrepancy between the proportion of transfeminine people and their underrepresentation in research is a critical gap in knowledge because transfeminine people, particularly transfeminine people of color, experience unique physical and mental health challenges[26-29].” (lines 58-64)

**ABSTRACT**

1. Reviewer #3: In response to other reviewers, the authors made edits in lines 4-7. The edits more clearly frame factors related DEB among TNG youth. I would encourage the authors to move away from the use of "transphobic discrimination" to "cissexism", as the latter term explicitly names the system of oppression driving discrimination against trans individuals and not a phobia of trans people.
   a. Thank you for suggesting a more up to date term. We have replaced “transphobic discrimination” with “cissexism” in line 7.

**MATERIALS AND METHODS**

Methods

1. Reviewer #3: The authors added information about payment for each phase of the study. Phase 1 notes that the YAB will be paid $250 for every 6 months of participation. The authors are asked to provide rationale for compensating members of the YAB in a lump-sum every 6 months as opposed to after every meeting, especially given the prevalence of financial disparities for trans youth and the length of time between meetings and payment. The authors are also asked to consider implications for retention of YAB members and how financial barriers may result in a lack of diversity among the YAB if compensation for time provided is only awarded twice per year.
   a. Thank you for this important comment. While our first resubmission was under review, the research team met with community partners and other researchers with TNG YAB experience to discuss and further refine our YAB compensation plan. After receiving feedback, we decided to provide more frequent compensation. We will be providing $125 for every 3 months of participation instead of $250 for every 6 months of participation. We are not providing compensation after each meeting because members may not be able to attend every meeting. Additionally, after receiving feedback, we will be providing $20 for each transcript that YAB members code in Phases 2 and 3. These changes are described in lines 92-95.
2. Reviewer #3: I thank the authors for adding information about protecting interviewees. I would encourage the authors to provide any additional information related to planned recording of the interviews, methods and timeline for transcription of recordings, and how/when interview transcripts will be de-identified and unlinked from recordings, as recordings carry the highest level of risk for participants.
   a. Thank you for the opportunity to add additional details to our data collection for qualitative interviews in Phases 2 and 3. We have added lines 217-221 and 224-227.

Inclusion and exclusion criteria

1. Reviewer 3: Thank you for this update. The authors are still asked to respond to how studies reviewed in the introduction that focus on minors apply to TNG individuals who are 18-21 and any additional considerations for this age group that is to be included in the study.
   a. Most of the studies cited in the introduction describe TNG youth, including participants up to 25 years of age. Other than consent processes, we do not have different protocols for participants 18 and older. For each phase, we are measuring age and thus, our findings may result in additional considerations for different age groups within TNG youth.

Data collection

1. Reviewer #3: I would encourage the authors to continue considering the need for collection of sex in Phase 4, in combination with Reviewer 1's suggestion to collect data regarding intersex status. Although measurement invariance is important, it remains unclear to me if asking participants about their sex assigned at birth is meaningful enough to offset potential impacts and not honor community-based recommendations. Will testing invariance by sex be limited to a male/female binary, and if so, how do the authors plan to include intersex individuals? Finally, do the authors have other theoretical rationale for including sex assigned at birth aside from capacity to menstruate? Due to variation in onset of puberty, use of birth control, and other factors, not all individuals who are assigned female at birth can or do menstruate. If the concern about measurement variance is only tied to menstruation, I would encourage authors to consider how to collect information about menstruation specifically.
   a. We thank the reviewer for their continued advocacy on affirming research procedures. We will be working closely with our community consultant, YAB, and community stakeholders for each Phase. Prior to Phase 4 data collection, we will assess the need to collect sex assigned at birth. We will follow the reviewer’s recommendations of considering the rationale, impacts, and inclusion of intersex people when discussing sex assigned at birth data collection and analysis with community members. We have added lines 257-258 to reflect that the list of measures will be finalized after receiving feedback from the community.
